# Monocytes reprogrammed by tumor microparticle vaccine inhibit tumorigenesis and tumor development

**DOI:** 10.1186/s12645-023-00190-x

**Published:** 2023-04-17

**Authors:** Weiwei Sun, Lili Dai, Yuqing Cao, Pengtao Pan, Lijuan Zhi, Xinke Wang, Xinzhong Yuan, Zi Gao, Sheng Guo, Guoyan Liu, Junlei Yin, Liangliang Xie, Liping Wang, Yanling Wang, Wensheng Li, Hong Li, Yunjie Jia

**Affiliations:** 1 School of Medicine, Xinxiang University, Jinsui Road 191, Xinxiang, 453003 China; 2 Department of Immunology, School of Basic Medical Sciences, Xinxiang Medical University, Xinxiang, China

**Keywords:** Tumor microparticles, Monocytes, Monocyte-derived DCs, IRF4, Tumor vaccine

## Abstract

Tumor microparticles (T-MPs) are considered as a tumor vaccine candidate. Although some studies have analyzed the mechanism of T-MPs as tumor vaccine, we still lack understanding of how T-MPs stimulate a strong anti-tumor immune response. Here, we show that T-MPs induce macrophages to release a key chemotactic factor CCL2, which attracts monocytes to the vaccine injection site and enhances endocytosis of antigen. Monocytes subsequently enter the draining lymph node, and differentiate into monocyte-derived DCs (moDCs), which present tumor antigens to T lymphocytes and deliver a potent anti-tumor immune response. Mechanically, T-MPs activate the cGAS-STING signaling through DNA fragments, and then induce monocytes to upregulate the expression of IRF4, which is a key factor for monocyte differentiation into moDCs. More importantly, monocytes that have endocytosed T-MPs acquire the ability to treat tumors. Collectively, this work might provide novel vaccination strategy for the development of tumor vaccines and facilitate the application of T-MPs for clinic oncotherapy.

**Supplementary Information:**

The online version contains supplementary material available at 10.1186/s12645-023-00190-x.

## Introduction

At present, the traditional treatment methods such as surgery, radiotherapy, and chemotherapy encounter a bottleneck period in the treatment of tumor. More than one century ago, William Coley discovered that microbes could be used as a stimulator for the immune system to treat cancer (Temizoz et al. 2016). Nowadays, immune checkpoint inhibitors through activation the patient's immune system against caner have been widely used in clinical practice (Yi et al. 2022). Numerous direct evidences have shown that the anti-tumor immune response plays a key role in oncotherapy (Meric-Bernstam et al. 2021; Versluis et al. 2020). The essence of anti-tumor immune response is that antigen-presenting cells (APCs) take up tumor antigens and present them to CD8^+^ T lymphocytes. Then, CD8^+^ T lymphocytes are activated, differentiate toward cytotoxic T lymphocytes, and own the ability of targeting and killing tumor cells (van der Leun et al. 2020). To this end, people try to find a breakthrough in tumor prevention and treatment through vaccines. Although a variety of tumor vaccines have entered the clinical trial stage, none of them achieve ideal results. The reasons are mainly as follows: (i) tumor vaccines cannot be effectively taken up and processed by APCs; (ii) APCs fail to be fully activated; (iii) tumor-specific T lymphocytes are not able to be sufficiently activated (Saxena et al. 2021; Wculek et al. 2020).

Apoptotic cells can release extracellular vesicles (EVs) that range in size between molecules and cells (Buzas 2022). Microparticles (MPs) are a type of EVs, which are formed by the cytomembrane encapsulating a part of cytosolic contents when cell receives stimuli or apoptosis signal. The diameter of MPs is between 100 and 1000 nm (Ma et al. 2021). Previous study showed that *L. monocytogenes* antigens were contained in MPs released by *L. monocytogenes*-infected macrophages. These MPs can induce the body to produce T-lymphocyte immune response against the bacteria, revealing that MPs have the potential to be vaccine (Zhang et al. 2012). Further studies found that when mice are immunized with tumor microparticles (T-MPs), the T-MP-mediated anti-tumor immune response can effectively prevent tumorigenesis (Dong et al. 2017; Zhang et al. 2015), indicating that T-MPs are a new type of tumor vaccine.

Suitable antigen and body's immune response against the antigen are two key factors in vaccine development. At present, the commonly used vaccines are composed of two parts: antigen and adjuvant. The function of adjuvant is to stimulate the body's immune response to antigen and enhance the efficacy of vaccines in preventing various diseases (Kobiyama and Ishii 2022). Our previous research showed that T-MPs contain tumor antigens which can be presented to T lymphocytes by dendritic cells (DCs) (Ma et al. 2018). Meanwhile, T-MPs can induce more DCs to enter the vaccine injection site and promote the maturation of DCs (Dong et al. 2017; Zhang et al. 2015). These studies indicated that T-MPs have both tumor antigen and function of adjuvant, indicating that T-MPs are an ideal tumor vaccine candidate (Ma et al. 2020). However, as a new type of tumor vaccine, the effect of T-MPs on the immune microenvironment at vaccine injection site is still unclear and needs to be clarified.

Our previous researches found that DCs play a key role in T-MPs as tumor vaccine (Dong et al. 2017; Ma et al. 2018; Zhang et al. 2015). There are two main types of DCs in lymph nodes of mice, namely classical DCs (cDCs) and monocyte-derived DCs (moDCs). Both moDCs and cDCs express CD11c and MHCII (Tussiwand and Rodrigues, 2020). moDCs derived from monocytes also express the monocyte-specific marker CD64, so CD11c^+^MHCII^+^CD64^+^ triple-positive cells are labeled as moDCs (Langlet et al. 2012; Lee et al. 2021). The number of DCs in draining lymph node (dLN) from mice immunized with T-MPs was found to be significantly increased. One possible reason is that cDCs in the surrounding tissue are attracted to the dLN. Another reason may be that monocytes enter the dLN and differentiate into moDCs after endocytosis of antigens. In this research, we elucidate the effects of T-MPs as tumor vaccine on the immune microenvironment at injection site, providing a vaccination therapy for the application of T-MPs.

## Results

### T-MPs are mainly taken up by monocytes and macrophages

**Fig. 1:**
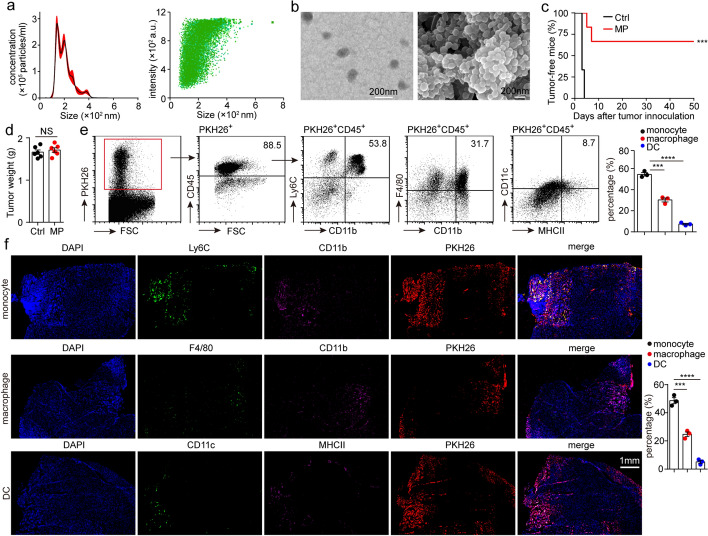
T-MPs are mainly taken up by monocytes and macrophages. **a** The size of H22-MPs was analyzed by NTA. **b** The morphology of H22-MPs was characterized by transmission and scanning electron microscopy. **c** BALB/c mice (*n* = 6), intramuscularly inoculated with PBS or H22-MPs on days -14, -13, and -7, were given i.m. injection of 3 × 10^5^ H22 hepatocarcinoma cells into the right rear thigh muscle on day 0. Kaplan–Meier analysis was used to assess the percentage of mice which were tumor-free. **d** Nude mice (*n* = 6) were intramuscularly immunized with PBS or H22-MPs on days -14, -13, and -7, and then mice were given i.m. inoculation with 3 × 10^5^ H22 hepatocarcinoma cells into the right rear thigh muscle on day 0. The tumor weight was measured on day 7. **e** BALB/c mice were intramuscularly inoculated with PKH26-labeled H22-MPs. After 24 h, CD45^+^ immunocytes in PKH26^+^ cells from thigh muscle were analyzed by flow cytometry. Meanwhile, CD11b^+^Ly6C^+^ monocytes, CD11b^+^F4/80^+^ macrophages, and CD11c^+^MHCII^+^ DCs in PKH26^+^CD45^+^ cells were also analyzed by flow cytometry. **f** BALB/c mice were intramuscularly inoculated with PKH26-stained H22-MPs. After 24 h, the thigh muscle tissues were removed for frozen sections and immunofluorescence staining, then observed by a two-photon confocal microscope. PKH26^+^ monocytes, PKH26^+^ macrophages, and PKH26^+^ DCs in total PKH26^+^ cells were analyzed by counting of fluorescent spots. Mean ± s.e.m. is represented in the data. Log-rank (**c**) or two-tailed unpaired Student's *t* test (**d–f**) was used to statistically analyze the *P* values. ***P < 0.001; ****P < 0.0001; NS, not significant

In a previous study, mice were subcutaneously immunized with T-MPs three times, and then were given subcutaneous (s.c.) inoculation with tumor cells. The results showed that s.c. vaccination of T-MPs effectively inhibited tumorigenesis (Zhang et al. 2015). Consistently, mice were immunized three times with T-MPs by oral delivery, followed by s.c. inoculation with tumor cells. The oral vaccination of T-MPs also significantly prevented tumor growth (Dong et al. 2017). Both studies demonstrated that T-MPs can be used as tumor vaccine. To further elucidate the mechanism of T-MPs as tumor vaccine, we immunized mice using intramuscular (i.m.) injection. H22 hepatocarcinoma cell-derived MPs (H22-MPs) were isolated from the supernatants of UV-irradiated H22 cells by centrifugation. Nanoparticle tracking analysis (NTA) showed that the size of H22-MPs was mostly around 140 nm (Fig. 1a), which was further affirmed by scanning and transmission electron microscopy (Fig. 1b). BALB/c mice were intramuscularly inoculated with H22-MPs, and then subjected to an injection of 3 × 10^5^ H22 hepatocarcinoma cells. The result showed that 100% tumorigenesis was seen in control group, whereas 66.7% of T-MP-immunized mice could prevent H22 tumor (Fig. 1c), suggesting that i.m. injection of H22-MPs could still effectively inhibit tumorigenesis of H22 hepatocarcinoma. To determine that immunogenicity of T-MPs by i.m. injection is not limited to one type of tumor, we intramuscularly inoculated C57BL/6 mice with B16-F10 melanoma cell-derived MPs (B16-MPs). Vaccinated mice could effectively prevent tumorigenesis of B16-F10 melanoma (Additional file 1: Fig. S1). Similarly, BALB/c mice intramuscularly inoculated with CT26 colon carcinoma cell-derived MPs (CT26-MPs) inhibited CT26 tumor growth (Additional file 1: Fig. S2). However, BALB/c mice immunized with H22-MPs via i.m. injection could not prevent tumorigenesis of CT26 tumor (Additional file 1: Fig. S2), suggesting that the anti-tumor immune response caused by T-MPs is specific immunity rather than innate immunity. Meanwhile, T-MPs as a tumor vaccine presented a good safety, evidenced by unaltered weight and normal function of the liver and kidney in mice after T-MP treatment (Additional file 1: Fig. S3). In addition, we found that H22-MPs were not able to elicit the aforementioned anti-tumor effect in T-lymphocyte-deficient BALB/c nude mice (Fig. 1d), suggesting that anti-tumor immunity induced by T-MPs is T-lymphocyte dependent.

To analyze which cells endocytosed T-MPs, mice were intramuscularly immunized with PKH26-stained H22-MPs. After 24 h, the muscles around vaccine injection site were isolated, and labeled with anti-CD45 flow antibody after digestion. The proportions of CD45^+^ and CD45^−^ cells in PKH26^+^ cells were analyzed by flow cytometry. The result showed that more than 85% of PKH26^+^ cells were CD45^+^ cells (Fig. 1e), suggesting that the cells which take up T-MPs are mostly immune cells. We then turned our attention to which immune cells endocytosed T-MPs. We further analyzed the endocytosis of T-MPs by immune cells in muscles via flow cytometry and immunofluorescence staining. The results indicated that T-MPs were mainly endocytosed by monocytes (53.8%) and macrophages (31.7%), and a small amount of T-MPs were endocytosed by DCs (8.7%) (Fig. 1e, f), while B lymphocytes and T lymphocytes did not endocytose T-MPs (Additional file 1: Fig. S4). We then immunized C57BL/6 mice with PKH26-stained B16-MPs. Similarly, B16-MPs were also mainly endocytosed by monocytes (52.8%) and macrophages (33.6%), and a small quantity of B16-MPs were endocytosed by DCs (6.7%), while B lymphocytes and T lymphocytes did not take up B16-MPs (Additional file 1: Fig. S5). Collectively, these data indicate that T-MPs are mainly taken up by monocytes and macrophages after mice are immunized with T-MPs.

### T-MPs draw monocytes in blood to the vaccine injection site

**Fig. 2:**
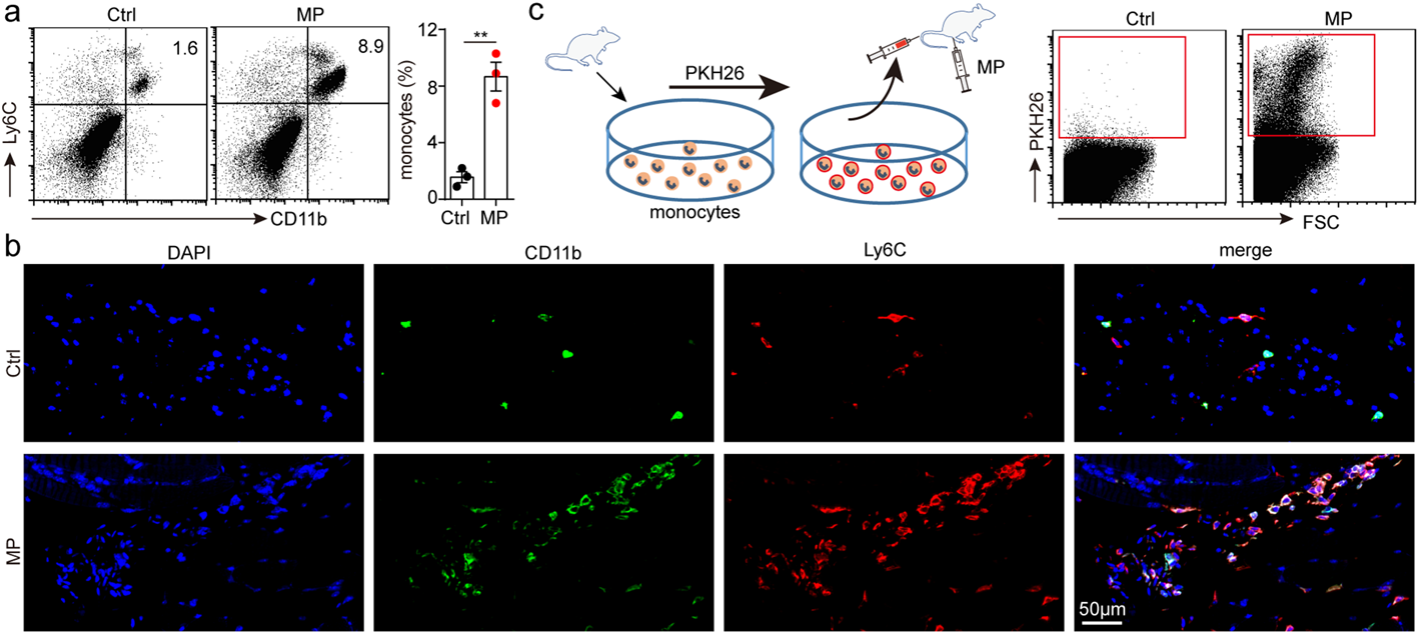
T-MPs draw monocytes in blood to the vaccine injection site. **a** BALB/c mice (*n* = 3) were intramuscularly immunized in the right rear thigh with H22-MPs and in the left rear thigh with PBS. After 48 h, mice were sacrificed, and monocytes in muscles around the femur were measured by flow cytometry. **b** Similar setting as in **a** and the thigh muscle tissues were removed for paraffin sections and immunofluorescence staining, then observed by a two-photon confocal microscope. **c** BALB/c mice were injected with 1 × 10^6^ PKH26-stained allogeneic monocytes by tail vein, followed by inoculation with H22-MPs via i.m. injection. After 48 h, recruitment of PKH26^+^ monocytes was analyzed via flow cytometry. Mean ± s.e.m. is represented in the data and two-tailed unpaired Student's *t* test was used to statistically analyze the *P* value. **P < 0.01

After the vaccine is injected into body, it is mainly endocytosed by macrophages which are professional phagocytic cells (Vesikari et al. 2018). However, in this study, we surprisingly found that the largest number of immune cells that take up T-MPs are monocytes. Further study found that the proportion of monocytes in immunized thigh was obviously higher than that of the opposite side (Fig. 2a, b). Normally, monocytes mainly circulate in blood (Guilliams et al. 2018). We wondered whether the monocytes in blood migrate to vaccine injection site. To analyze the origin of increased monocytes, we injected PKH26-stained allogeneic monocytes into mice by tail vein, then immunized mice with H22-MPs via i.m. injection, and analyzed the cells at vaccine injection site 48 h later. We found that there were many PKH26^+^ cells derived from blood at the vaccine injection site (Fig. 2c), which indicated that the increased monocytes were mainly derived from blood.

### T-MPs induce CCL2 upregulation in macrophages

**Fig. 3:**
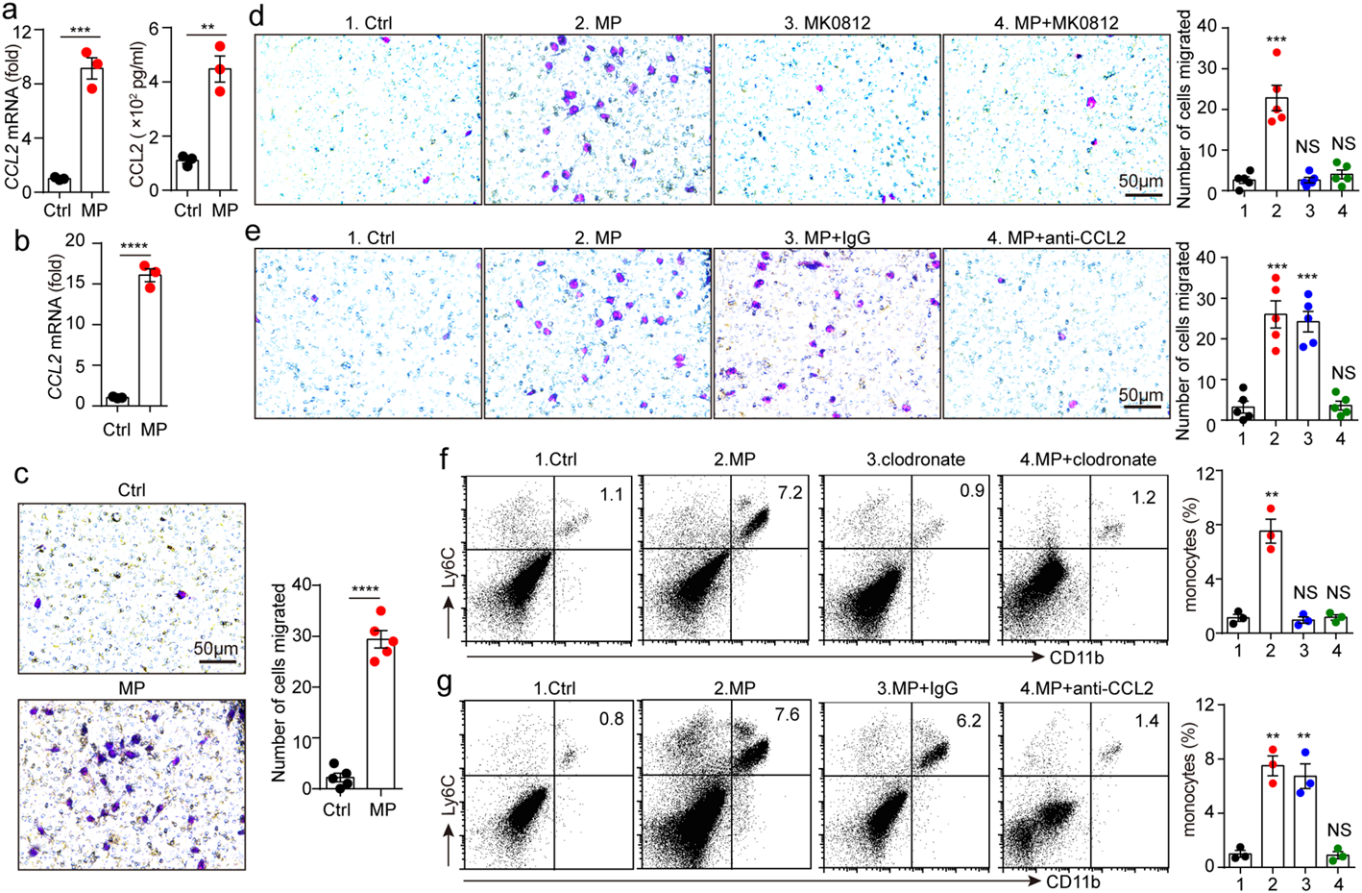
T-MPs induce CCL2 upregulation in macrophages. **a** BALB/c mouse peritoneal macrophages were cultured with H22-MPs. After 24 or 72 h, RNAs or cultured medium was collected respectively. Then, the expression of CCL2 was measured using real-time PCR or ELISA. **b** BALB/c mice were intramuscularly inoculated with H22-MPs or PBS. 24 h later, CCL2 expression of isolated monocytes from thigh muscles was measured by real-time PCR. **c** BALB/c mouse bone marrow-derived macrophages (BMDMs) were co-incubated with H22-MPs in a 24-well plate, and isolated monocytes from bone marrow cells were cultured in the upper chamber, followed by monocyte migration assay after 48 h. **d** BALB/c mouse BMDMs were co-incubated with H22-MPs, and monocytes were cultured with or without CCR2 antagonist MK0812 in the upper chamber, followed by monocyte migration assay after 48 h. **e** BALB/c mouse BMDMs were treated with H22-MPs and CCL2 neutralizing antibody, and monocytes were cultured in the upper chamber, followed by monocyte migration assay after 48 h. **f** BALB/c mice were subjected to clodronate liposomes by tail vein twice a week for 3 weeks, then mice were treated with H22-MPs via i.m. injection. After 48 h, the proportion of monocytes in muscles around the femur was measured by flow cytometry. **g** BALB/c mice were given i.m. injection with H22-MPs, PBS control or intraperitoneally pretreatment with anti-CCL2 neutralizing antibody. After 48 h, the proportion of monocytes in muscles around the femur was measured via flow cytometry. All of experimental groups compared with control group. Mean ± s.e.m. is represented in the data and two-tailed unpaired Student's *t* test was used to statistically analyze the *P* values. **P < 0.01; ***P < 0.001; ****P < 0.0001; NS, not significant

CCL2 is the most important chemokine for chemotaxis of monocytes (Jakubzick et al. 2017). Our previous researches showed that after macrophages endocytosed T-MPs, the expression of CCL2 and other chemokines could be upregulated (Dong et al. 2017; Zhang et al. 2018). Here, we wondered whether the increased monocytes at vaccine injection site related to the secretion of CCL2 by macrophages. We detected the expression of CCL2 after co-incubating mouse peritoneal macrophages with T-MPs and found that CCL2 expression was obviously upregulated (Fig. 3a). To directly confirm that T-MPs induce CCL2 upregulation in macrophages at the vaccine injection site, we isolated macrophages in muscles and detected the expression of CCL2. The results showed that CCL2 expression was upregulated (Fig. 3b). Subsequently, we used trans-well chambers to explore whether macrophages could draw monocytes by secreting CCL2 after uptake of T-MPs. The results showed that macrophages could attract monocytes to the lower chamber after endocytosis of T-MPs (Fig. 3c). We added CCR2 antagonist MK0812 to the upper chamber, or CCL2 neutralizing antibody to the lower chamber, and repeated the above experiment. The chemotactic effect of monocytes was significantly reduced (Fig. 3d, e). Therefore, we speculated that macrophages that have endocytosed T-MPs promote monocyte migration to the vaccine injection site by secreting CCL2. To further analyze the chemotaxis of monocytes after macrophage uptake of T-MPs, mice were intravenously injected with clodronate liposomes to deplete macrophages, and then were given i.m. administration of T-MPs. Finally, the proportions of monocytes at vaccine injection site were measured via flow cytometry. The results indicated that in the absence of macrophages endocytosing T-MPs, there was no significant increase in monocytes at the vaccine injection site (Fig. 3f). To analyze whether CCL2 secreted by macrophages attracts monocytes to the vaccine injection site, we first injected mice intraperitoneally with CCL2 neutralizing antibody, and then immunized the mice with T-MPs by i.m. injection. After 48 h, the proportions of monocytes at vaccine injection site were measured via flow cytometry. The results showed that the proportion of monocytes in the isotype control group was significantly increased, which was consistent with the group inoculated with T-MPs alone, while the proportion of monocytes in the CCL2 neutralizing antibody group did not change, which was similar to the PBS control group (Fig. 3g). Together, these data indicate that T-MPs injected into mice are first endocytosed by macrophages, which secrete CCL2 and draw monocytes to the vaccine injection site.

### Monocytes that have endocytosed T-MPs enter dLN and differentiate into moDCs

**Fig. 4:**
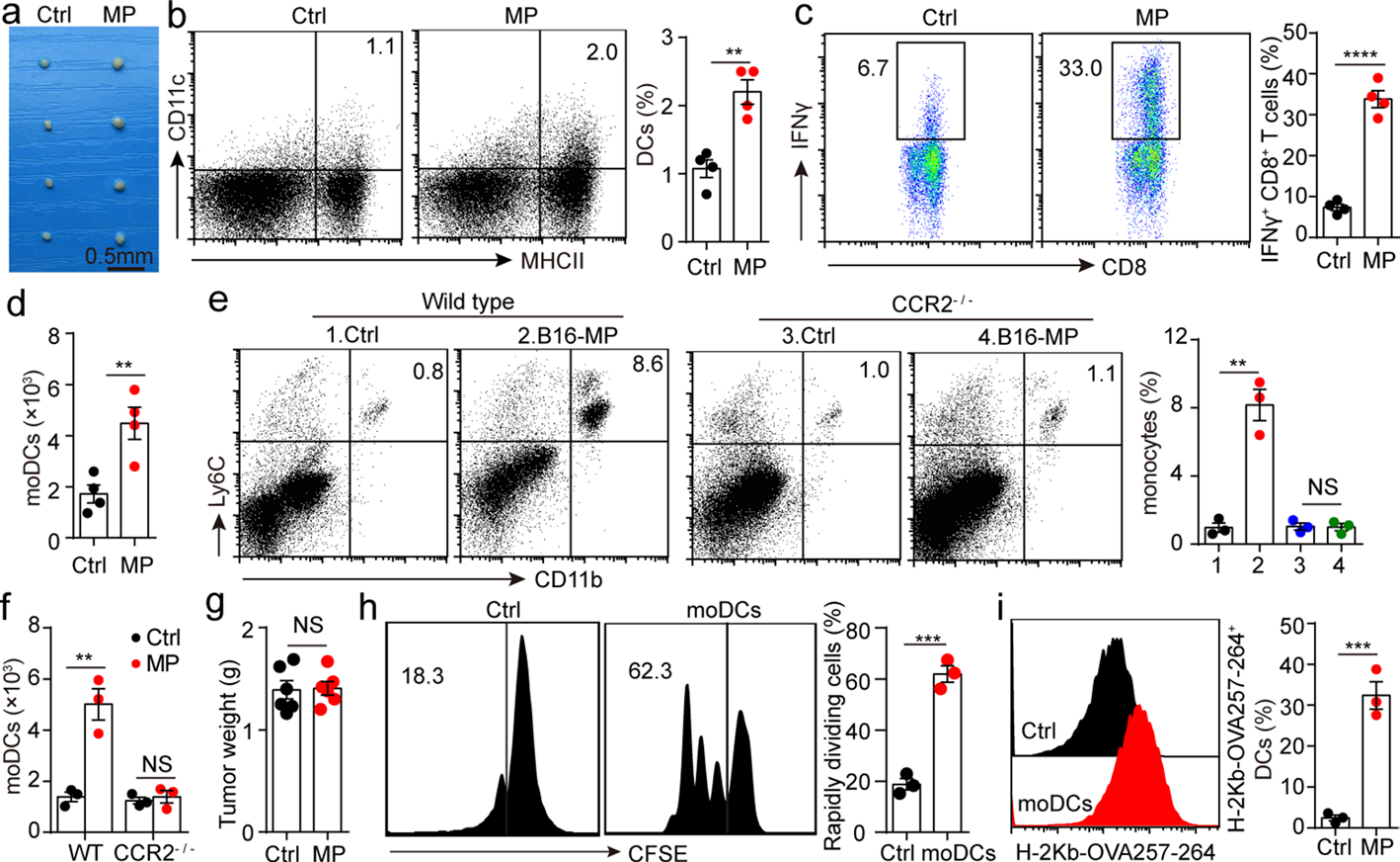
Monocytes that have endocytosed T-MPs enter dLN and differentiate into moDCs. **a–d** BALB/c mice (*n* = 4) were immunized in the right rear thigh with H22-MPs and in the left rear thigh with PBS via i.m. injection. After 48 h, mice were sacrificed, and inguinal lymph nodes of both legs were isolated which were taken picture with a digital camera (**a**). DCs (**b**), CD8^+^IFNγ^+^ T lymphocytes (**c**) and moDCs (**d**) in inguinal lymph nodes were measured via flow cytometry. **e** Wild-type (WT) C57BL/6 mice and CCR2^−/−^ mice were intramuscularly injected with B16-MPs. After 48 h, monocytes in thigh muscles were measured by flow cytometry. **f** WT C57BL/6 mice and CCR2^−/−^mice were immunized in the right rear thigh with B16-MPs and in the left rear thigh with PBS via i.m. injection. After 48 h, mice were sacrificed, and moDCs in inguinal lymph nodes were measured via flow cytometry. **g** CCR2^−/−^ mice immunized with B16-MPs or PBS were subjected to an i.m. injection of 5 × 10^5^ B16-F10 melanoma cells on day 0. The tumor weight was measured on day 10. **h** Splenic CD8^+^ T lymphocytes derived from OT-1 mice were co-incubated with CD11c^+^MHCII^+^CD64^−^ DCs (Ctrl) or CD11c^+^MHCII^+^CD64^+^ moDCs from OVA-MP immunized C57BL/6 mice. T-lymphocyte proliferation was measured using flow cytometry. **i** The expression of the H-2 Kb-OVA257-264 peptide complex in CD11c^+^MHCII^+^CD64^−^ DCs (Ctrl) and CD11c^+^MHCII^+^CD64^+^ moDCs was measured via flow cytometry. Mean ± s.e.m. is represented in the data and two-tailed unpaired Student's *t* test was used to statistically analyze the *P* values. **P < 0.01; ***P < 0.001; ****P < 0.0001; NS, not significant

We have found that i.m. injection of T-MPs produces anti-tumor immune response that relies on T lymphocytes, while T lymphocytes do not endocytose T-MPs, suggesting that engulfment of T-MPs and antigen presentation by APCs are critical for anti-tumor immunity. Although there are different types of APCs at thigh muscles, DCs are generally considered as the professional APCs that are indispensable for stimulating adaptive immunity (Bošnjak et al. 2022). Most of the monocytes in the body exist in blood, and a part of monocytes in blood can migrate toward lymph nodes to differentiate into monocyte-derived DCs (moDCs) and stimulate the specific proliferation of T lymphocytes (Kurup et al. 2019). To analyze the role of moDCs in anti-tumor immunity, we isolated the dLNs from mice immunized with T-MPs and found they became significantly larger (Fig. 4a), and the proportions of DCs and CD8^+^IFNγ^+^ T lymphocytes were significantly increased (Fig. 4b, c). We further analyzed the number of CD11c^+^MHCII^+^CD64^+^ moDCs in dLN, and discovered that the number of moDCs was obviously increased (Fig. 4d). moDCs are normally considered as nonproliferating cells (Gardner and Ruffell, 2018). We did not discover that T-MP treatment lead to the proliferation of CD11c^+^MHCII^+^CD64^+^ cells in the dLN by using the proliferation marker Ki-67 (Additional file 1: Fig. S6), suggesting that monocytes derived from the blood differentiated toward moDCs. To provide more direct evidence for this hypothesis, we intramuscularly immunized CCR2^−/−^ mice with B16-MPs, and discovered that the recruitment of monocytes to vaccine injection site disappeared (Fig. 4e). Consistently, T-MPs neither promoted moDCs increase in dLN from CCR2^−/−^ mice (Fig. 4f), nor triggered anti-tumor immune response (Fig. 4g). Here, we wondered whether the moDCs differentiated from monocytes had endocytosed T-MPs. C57BL/6 mice were intramuscularly immunized with B16-OVA melanoma cell-derived MPs (OVA-MPs). After 48 h, dLNs were isolated, and then CD11c^+^MHCII^+^CD64^+^ and CD11c^+^MHCII^+^CD64^−^ cells were isolated by flow sorting, which were cultured with OT-1 CD8^+^ T lymphocytes, respectively. We found that CD11c^+^MHCII^+^CD64^+^ moDCs could promote CD8^+^ T lymphocyte proliferation more effectively than CD11c^+^MHCII^+^CD64^−^ DCs (Fig. 4h). Further, we analyzed the expression of the H-2Kb-OVA257-264 peptide complex in both types of DCs and found that a larger proportion of moDCs expressed the H-2Kb-OVA257-264 peptide complex (Fig. 4i), which explained CD11c^+^MHCII^+^CD64^+^ moDCs could more effectively promote CD8^+^ T lymphocyte proliferation. Together, these results suggest that the monocytes which have endocytosed T-MPs differentiate into moDCs, present antigenic peptide to T lymphocytes, and stimulate the specific proliferation of T lymphocytes, thereby triggering a powerful anti-tumor immune response.

### T-MPs induce monocytes to upregulate the expression of IRF4

**Fig. 5:**
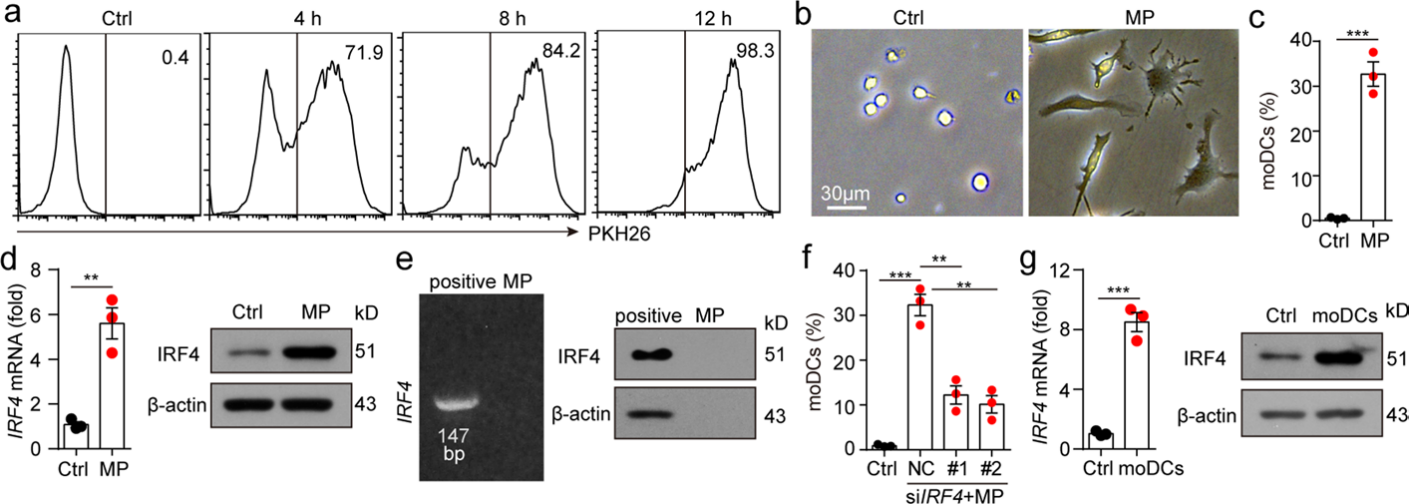
T-MPs induce monocytes to upregulate the expression of IRF4. **a** PKH26-labeled H22-MPs were co-incubated with BALB/c mouse monocytes for 4, 8, or 12 h, and the engulfment of H22-MPs by monocytes was measured by flow cytometry. **b**, **c** Monocytes and H22-MPs were co-incubated for 48 h, and the cell morphology was observed under an inverted microscope (**b**). The percent of CD11c^+^MHCII^+^CD64^+^ moDCs was measured via flow cytometry (**c**). **d** Monocytes were co-incubated with H22-MPs. After 24 or 48 h, RNA or protein were collected respectively. Then, the expression of IRF4 was measured at the levels of both gene and protein. **e** IRF4 mRNA and protein expression of H22-MPs was measured by RT-PCR and western blot. BALB/c mouse bone marrow-derived monocytes co-incubated with H22-MPs were served as positive controls for H22-MPs. **f** Monocytes transfected with NC siRNA or *IRF4* siRNA were cultured with H22-MPs for 48 h. The percent of CD11c^+^MHCII^+^CD64^+^ moDCs was analyzed by flow cytometry. **g** BALB/c mice were subjected to H22-MPs or PBS via i.m. injection. 24 or 48 h later, IRF4 expression of isolated monocytes from thigh muscles was analyzed at the levels of both gene and protein. Mean ± s.e.m. is represented in the data and two-tailed unpaired Student's *t* test was used to statistically analyze the *P* values. **P < 0.01; ***P < 0.001

To understand the mechanism during monocyte differentiation into moDCs, we examined the engulfment of T-MPs by monocytes in vitro. Monocytes and PKH26-labeled T-MPs were co-incubated for 4, 8, or 12 h, then PKH26-positive monocytes were measured by flow cytometry. The result showed that the engulfment of T-MPs by monocytes was obvious at 4 h (71.9%) and peaked at 12 h (98.3%) (Fig. 5a). To observe the effect of T-MPs on morphology of monocytes, T-MPs were co-incubated with monocytes for 48 h. The morphology of the cells was changed from round or oval to dendritic branches (Fig. 5b). Further, we analyzed the phenotype of the cells via flow cytometry and found that about 30% of the monocytes differentiated into CD11c^+^ MHCII^+^ CD64^+^ moDCs (Fig. 5c). The differentiation of monocytes into moDCs is mainly related to the transcription factor IRF4. Monocytes differentiate into moDCs when IRF4 expression is upregulated (Devalaraja et al. 2020; Goudot et al. 2017). To clarify the mechanism of monocytes differentiating into moDCs, we co-incubated monocytes with T-MPs for 24 or 48 h, and found the expression of IRF4 in the T-MP co-incubation group was upregulated at the levels of both gene and protein (Fig. 5d). This upregulation of IRF4 was not due to the IRF4 contained in T-MPs, since we didn't detected IRF4 mRNA or protein in T-MPs (Fig. 5e). To analyze whether IRF4 plays a decisive role in monocyte differentiation into moDCs, we knocked down IRF4 in monocytes which were co-incubated with T-MPs, and found that the differentiation of monocytes into moDCs was significantly inhibited (Fig. 5f). Consistently, compared with bone marrow-derived monocytes, moDCs from dLN upregulated IRF4 (Fig. 5g). Collectively, these results indicate that T-MPs can lead to monocyte differentiation toward moDCs via upregulation of IRF4 expression.

### DNAs in T-MPs stimulate monocytes to upregulate IRF4 expression

**Fig. 6:**
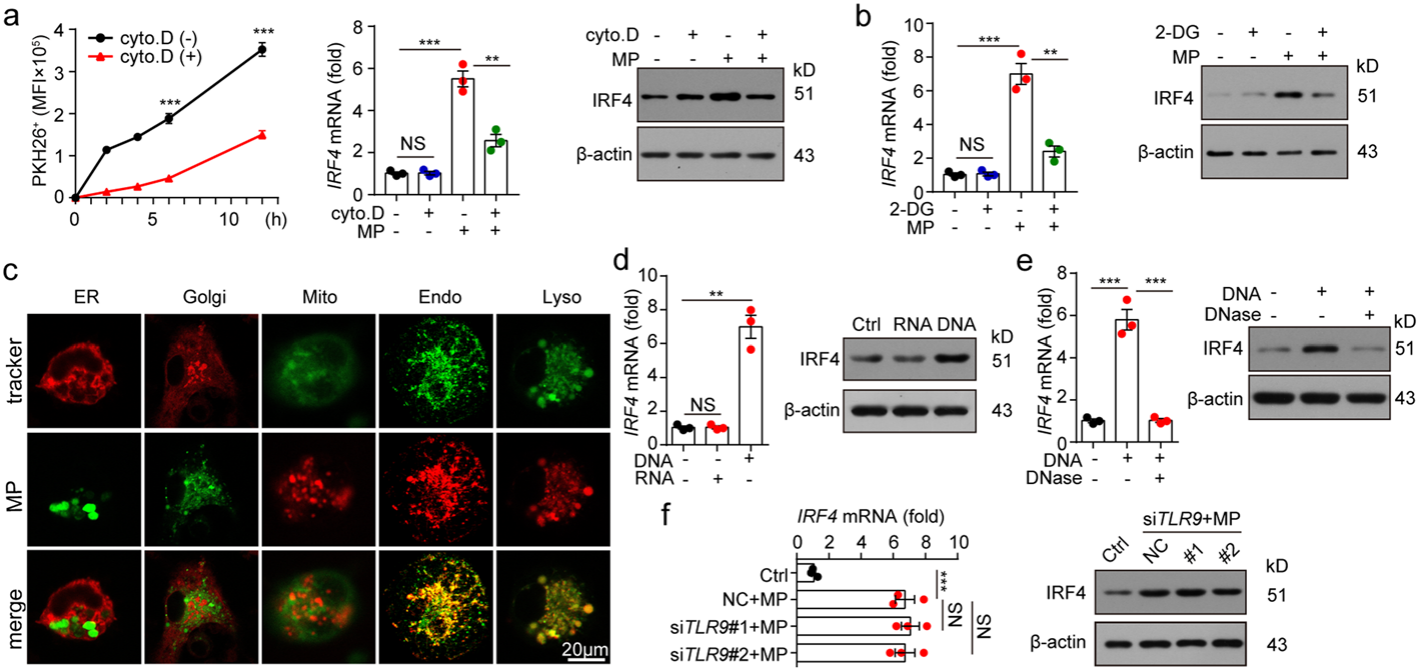
DNAs in T-MPs stimulate monocytes to upregulate IRF4 expression. **a** BALB/c mouse monocytes were co-incubated with PKH26-stained H22-MPs, cytochalasin D or PKH26-stained H22-MPs mixed with cytochalasin D. After 2, 4, 6 or 12 h, PKH26 fluorescence intensity of monocytes was measured via flow cytometry, and the IRF4 expression was analyzed at the levels of both gene and protein. **b** Monocytes pretreated with or without 2-DG for 30 min were co-incubated with H22-MPs for 24 h or 48 h. IRF4 expression was measured at the levels of both gene and protein. **c** Monocytes were co-cultured with PKH26 or PKH67-labeled H22-MPs for 14 h. Then the monocytes were analyzed with ER, Golgi, mitochondria, endosome or lysosome fluorescent trackers using a two-photon confocal microscope. **d** Monocytes were treated with DNAs or RNAs extracted from H22-MPs. After 24 or 48 h, the IRF4 expression was analyzed at the levels of both gene and protein. **e** Monocytes were co-cultured with H22-MP-derived DNAs or H22-MP-derived DNAs pretreated with DNase. 24 or 48 h later, IRF4 expression was analyzed at the levels of both gene and protein. **f** Monocytes transfected with *TLR9* siRNA were co-cultured with H22-MPs. After 24 or 48 h, IRF4 expression was analyzed at the levels of both gene and protein. Mean ± s.e.m. is represented in the data and two-tailed unpaired Student's *t* test was used to statistically analyze the *P* values. **P < 0.01; ***P < 0.001; NS, not significant

Monocytes are a class of cells with strong phagocytic ability (Shi and Pamer 2011). If we treated monocytes with cytochalasin D, an inhibitor of endocytosis, the uptake of T-MPs by monocytes was blocked, thereby inhibiting the upregulation of IRF4 expression (Fig. 6a). Endocytosis is an ATP-consuming process. Consistently, if we co-incubated monocytes with 2-deoxy-d-glucose (2-DG) to inhibit ATP production, it would also hinder IRF4 upregulation (Fig. 6b), indicating that endocytosis of T-MPs is necessary for IRF4 upregulation in monocytes. Consistent with this fact, fluorescent staining showed that T-MPs didn't localized in mitochondria, endoplasmic reticulum or Golgi apparatus, but were found to be colocalized with endosomes and lysosomes (Fig. 6c). Toll-like receptors are present in the endosomes, in which Toll-like receptor 3 (TLR3) recognizes the dsRNA, Toll-like receptor 7 (TLR7) and Toll-like receptor 8 (TLR8) recognize the ssRNA, and Toll-like receptor 9 (TLR9) recognizes the unmethylated CpG DNA (Fitzgerald and Kagan 2020). T-MPs carry various biologically active components of their parent cells, such as protein, lipid and nucleic acids, which are important means of signaling between cells (Tang et al. 2022). Our previous studies had shown that nucleic acids exist in T-MPs (Chen et al. 2020; Zhang et al. 2015). We speculated that nucleic acids in T-MPs may activate TLRs, which in turn lead to IRF4 upregulation. To explore whether nucleic acids in T-MPs can induce IRF4 upregulation, we isolated DNAs and RNAs from T-MPs (Additional file 1: Fig. S7) and used them to stimulate mouse bone marrow-derived monocytes. We found that DNAs from T-MPs induced IRF4 upregulation, but RNAs from T-MPs had no such function (Fig. 6d). In parallel, co-incubation with DNase to destroy the DNAs dissolved the upregulation of IRF4 (Fig. 6e), suggesting that DNAs in T-MPs induce the upregulation of IRF4. Since TLR9 binds DNA, we speculated that DNAs in T-MPs may induce IRF4 upregulation by activating TLR9. However, co-incubation of DNAs extracted from T-MPs with TLR9-deficient monocytes still induced IRF4 upregulation in monocytes (Fig. 6f), suggesting that TLR9 is not necessary for T-MPs to induce upregulation of IRF4.

### T-MPs promote IRF4 upregulation by activating the cGAS-STING pathway

**Fig. 7:**
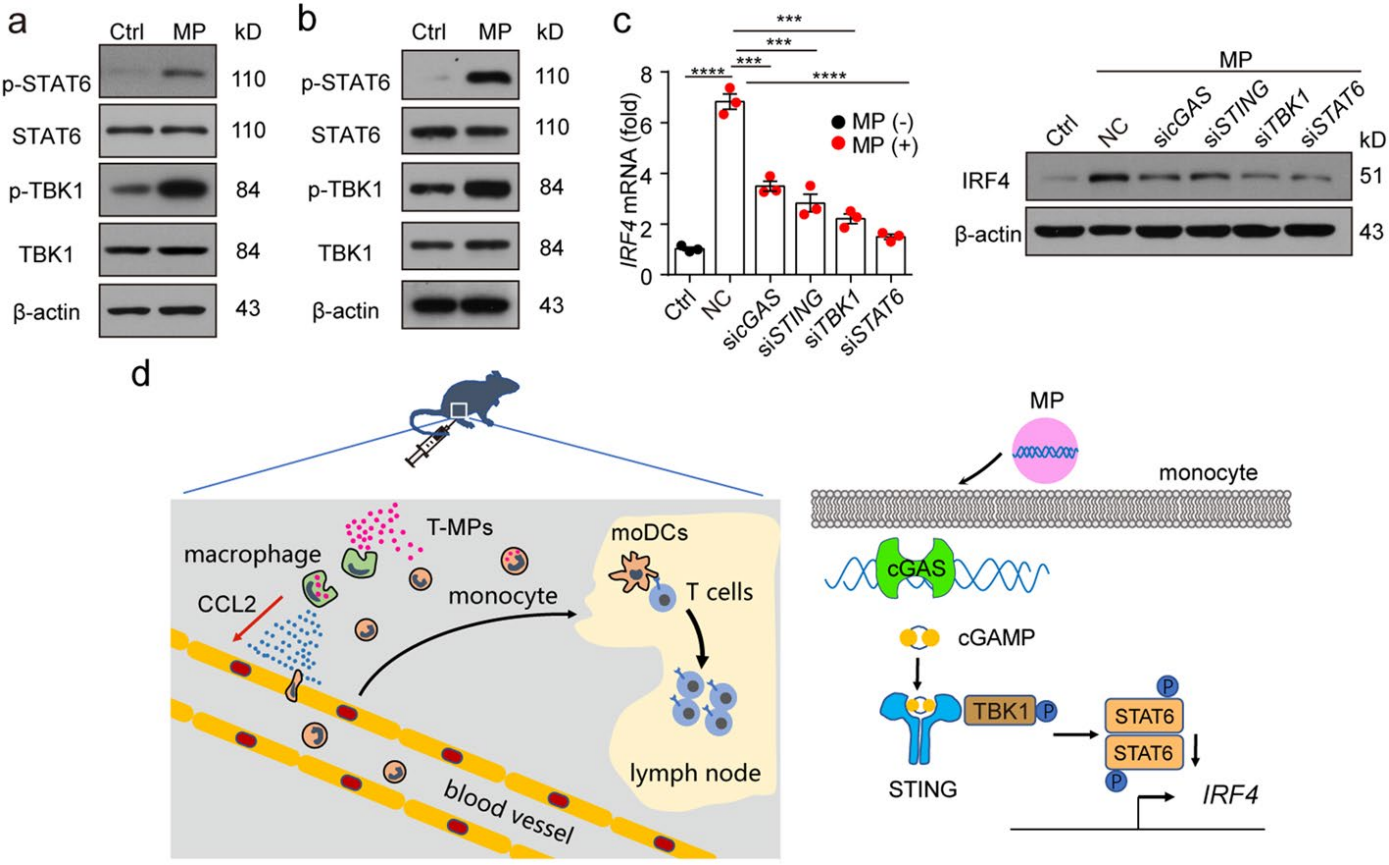
T-MPs promote IRF4 upregulation by activating the cGAS-STING pathway. **a** BALB/c mouse monocytes were co-cultured with H22-MPs for 24 h. Then the phosphorylation of TBK1 and STAT6 was measured by western blot. **b** BALB/c mice were subjected to an i.m. injection of PKH26-stained H22-MPs for 24 h. Then, CD11b^+^Ly6C^+^PKH26^+^ monocytes around thigh muscles were isolated by flow sorting, and the phosphorylation of TBK1 and STAT6 was detected by western blot. **c** Monocytes transfected with *cGAS*, *STING*, *TBK1* or *STAT6* siRNA were co-incubated with H22-MPs. After 24 or 48 h, IRF4 expression was analyzed at the levels of both gene and protein. **d** The schematic diagram of effects on the immune microenvironment at T-MP injection site (left), and the mechanism by which T-MPs induce monocytes to differentiate into moDCs (right). Mean ± s.e.m. is represented in the data and two-tailed unpaired Student's *t* test was used to statistically analyze the *P* values. ***P < 0.001; ****P < 0.0001

The next work is to explore the molecular mechanism of T-MP-induced IRF4 upregulation. Studies have shown that genomic and mitochondrial DNAs were contained in T-MPs from parental cells, which can activate the cGAS-STING-STAT6 signaling axis (Ma et al. 2016a; b; Zhang et al. 2015). Meanwhile, some researches have reported that IRF4 is a downstream effector of STAT6 (Goswami et al. 2012; Huang et al. 2016). To this end, we co-incubated monocytes with T-MPs, and detected the phosphorylation of TBK1 and STAT6 by western blot. As shown in Fig. 7a, phosphorylation of TBK1 and STAT6 in T-MP-treated monocytes was enhanced. Furthermore, we immunized mice with PKH26-stained T-MPs, isolated CD11b^+^Ly6C^+^PKH26^+^ monocytes which have endocytosed T-MPs in thigh muscle by flow sorting after 24 h, and detected the phosphorylation of TBK1 and STAT6 by western blot. Phosphorylation of TBK1 and STAT6 was still enhanced in T-MP-immunized group compared with PBS control group (Fig. 7b). In parallel, upregulated expression of IRF4 in monocytes was consistently inhibited using *cGAS*, *STING*, *TBK1* or *STAT6* siRNA (Fig. 7c), suggesting that DNAs within T-MPs contribute to the upregulation of IRF4 through the cGAS-STING-STAT6 signaling axis. Collectively, these results suggest that T-MPs promote IRF4 upregulation via the cGAS-STING signaling pathway.

### T-MP-loaded monocytes can achieve therapeutic effects

**Fig. 8:**
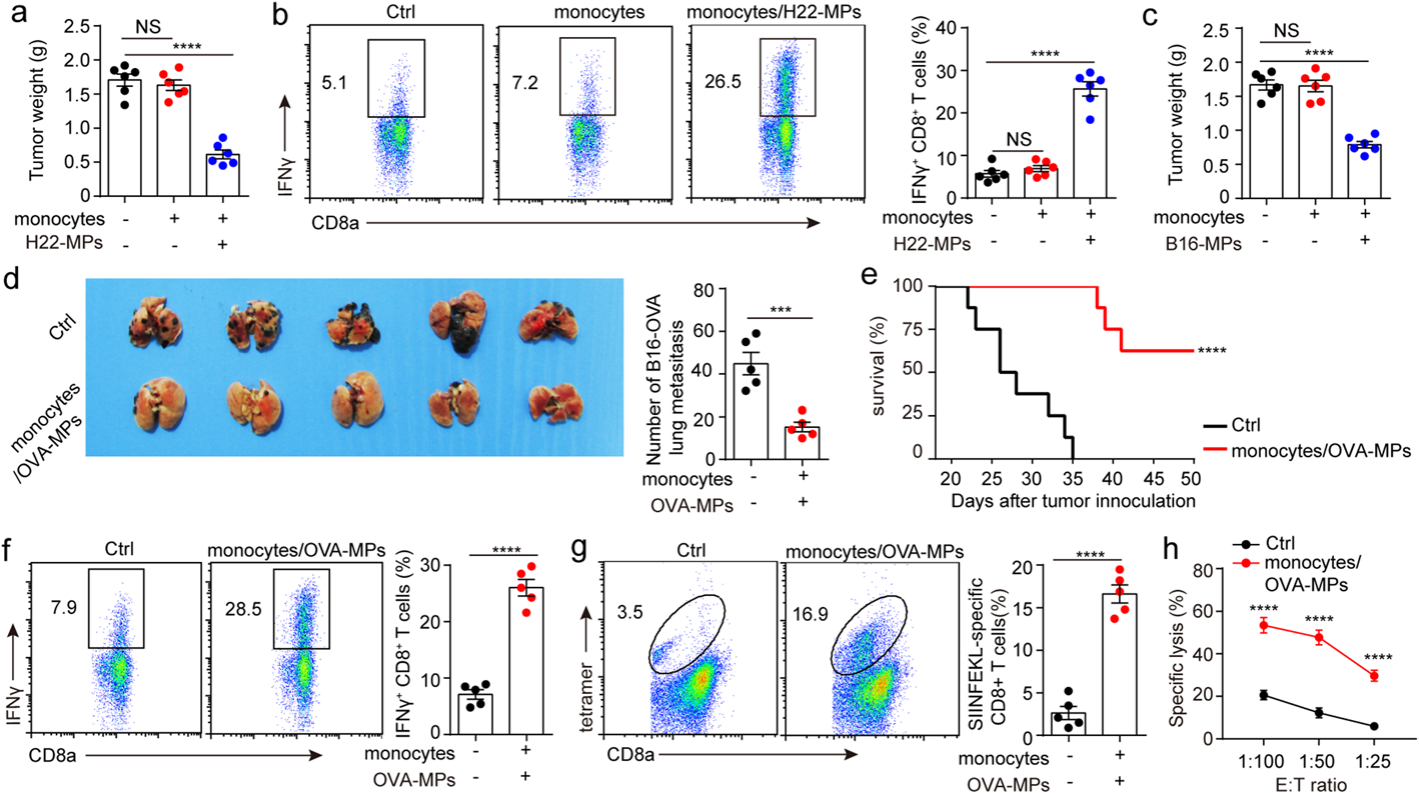
T-MP-loaded monocytes can achieve therapeutic effects. **a** BALB/c mice (*n* = 6) were preinoculated with 3 × 10^5^ H22 hepatocarcinoma cells in the right rear thigh on day 0. Then monocytes, H22-MP-loaded monocytes or PBS were injected into the mice through tail vein on day 5. The tumor weight was measured on day 10. **b** Infiltrating lymphocytes from the above tumors were collected and the proportion of CD8^+^IFNγ^+^ T lymphocytes was measured via flow cytometry. **c** Similar setting as in **a** but C57BL/6 mice (*n* = 6) were preinoculated with 5 × 10^5^ B16-F10 melanoma cells and then vaccinated with monocytes, B16-MP-loaded monocytes or PBS through intravenous injection. **d** C57BL/6 mice (*n* = 5) were preinoculated with 1 × 10^6^ B16-OVA tumor cells through tail vein on day 0. After 5 days, OVA-MP-loaded monocytes or PBS were injected into the mice through tail vein three times, once every 5 days. On day 21, the B16-OVA tumor nodules in lungs were calculated. **e** Similar setting as in **d** and mice survival were analyzed (*n* = 8). **f** and **g** Similar setting as in **d** and the proportions of CD8^+^IFNγ^+^ T lymphocytes and SIINFEKL-specific CD8^+^ T lymphocytes in spleen of mice (*n* = 5) were measured by flow cytometry 21 days after B16-OVA tumor inoculation. **h** Similar setting as in **d** and splenocytes were isolated on day 21. Splenocytes were treated with irradiated B16-OVA cells, and used as effector cytotoxic T lymphocytes in tumor-specific cytotoxicity assay after 5 days. Mean ± s.e.m. is represented in the data. Log-rank test (**e**) or two-tailed unpaired Student's *t* test was used to statistically analyze the *P* values. ***P < 0.001; ****P < 0.0001; *NS* not significant

We have shown that T-MPs prevent tumors by reprogramming monocytes. Here, we wondered whether T-MP-loaded monocytes can be used to treat tumors. To this end, BALB/c mice were subjected to 3 × 10^5^ H22 hepatocarcinoma cells in the right rear thigh via i.m. injection, and then vaccinated with 1 × 10^6^ H22-MP-loaded monocytes through intravenous injection 5 days after tumor inoculation. The result showed that vaccination with H22-MP-loaded monocytes obviously restrained H22 hepatocarcinoma growth. But in monocytes alone and PBS control group, we could not observe the tumor treatment effect (Fig. 8a). In parallel, the proportion of CD8^+^IFNγ^+^ T lymphocytes from tumor tissue in H22-MP-loaded monocytes group was obviously higher than that in the monocytes alone and PBS control group (Fig. 8b), suggesting that transplantation of monocytes alone may not lead to adequately activate T lymphocytes, possibly due to the fact that monocytes did not undergo reprogramming by T-MPs. Consistently, we found that B16-MP-loaded monocytes could inhibit B16-F10 melanoma growth (Fig. 8c). Using B16-OVA melanoma cell lung metastasis model, we further confirmed that transplantation of OVA-MP-loaded monocytes effectively treated B16-OVA cell lung metastasis (Fig. 8d) and prolonged the survival of the mice (Fig. 8e). In line with intravenous injection of T-MP-loaded monocytes, a plenty of IFNγ-producing SIINFEKL-H-2 Kb tetramer^+^CD8^+^ T lymphocytes were found (Fig. 8f, g), which efficiently cytolyzed B16-OVA cells (Fig. 8h). These results suggest that T-MP-loaded monocytes can improve the specific proliferation of CD8^+^ T lymphocytes, generating an effective anti-tumor consequence.

## Discussion

Although some researches have analyzed the mechanism of T-MPs as tumor vaccine, we still lack understanding of how T-MPs stimulate a potent anti-tumor immune response. In this research, we analyzed the effects of T-MPs on the immune microenvironment at vaccine injection site and illustrated in Fig. 7d: (i) T-MPs are first endocytosed by macrophages, which then upregulate CCL2; (ii) CCL2 recruits monocytes to vaccine injection site from circulating blood, and the endocytosis of T-MPs was enhanced by monocytes; (iii) DNAs contained in T-MPs induce monocytes to upregulate IRF4 by activating the cGAS-STING signaling pathway, thereby enabling monocytes to differentiate into moDCs; (iv) moDCs present tumor antigens to T lymphocytes, induce T lymphocyte-specific proliferation, and stimulate a strong anti-tumor immune response.

After vaccine is injected into body, it is mainly endocytosed by macrophages which are professional phagocytic cells (Vesikari et al. 2018). However, in this study, we found that the largest number of immune cells that engulfed T-MPs were monocytes, which were attracted to the vaccine injection site from blood when their surface receptor CCR2 bind to CCL2. The reason why T-MP injection site is rich in CCL2 is that macrophages upregulated CCL2 after endocytosing T-MPs.

Since the number of monocytes in body is much more than that of macrophages (Jung 2018), monocytes can enhance endocytosis of tumor antigens when they were attracted to T-MP injection site. Moreover, monocytes have the potential to differentiate into DCs (Robinson et al. 2021). In this study, we found that monocytes migrated to dLN and differentiated into moDCs, which explained the main reason for the increase of DCs in dLN after T-MPs were injected into mice. moDCs which have endocytosed T-MPs can present tumor antigens to CD8^+^ T lymphocytes, thereby stimulating the specific proliferation of T lymphocytes and inducing a potent anti-tumor immune response. This indicates that T-MPs have both tumor antigens and adjuvant efficacy, which make T-MPs an ideal tumor vaccine candidate. Predictably, if T-MPs and conventional adjuvant, such as alum or MF59 are combined, it may work better. In addition, we found that moDCs can stimulate the proliferation of CD8^+^ T lymphocytes more efficiently than other types of DCs. The reason may be that moDCs are differentiated from monocytes which have taken up T-MPs, so they can cross-present tumor antigens to CD8^+^ T lymphocytes more efficiently. However, the endocytosis of tumor antigens by other types of DCs is not sufficient.

The differentiation of monocytes toward DCs is mainly related to the upregulation of IRF4 (Devalaraja et al. 2020; Goudot et al. 2017). Here, we found that monocytes upregulate IRF4 and differentiate into moDCs after co-incubation with T-MPs. MPs are enriched with selective protein and nucleic acid from parental cells (Gao et al. 2020; Ma et al. 2016a, b; Xu et al. 2020). Moreover, MPs play an important role in delivering cellular components (Jin et al. 2017; Sun et al. 2017; Wang et al. 2022), and we found that T-MPs also deliver DNAs to stimulate monocytes to differentiate toward moDCs. In eukaryotic cells, DNAs are wrapped in the nucleus and mitochondria, avoiding direct contact with the cytoplasm (Fingerhut and Yamashita 2022). After microbial infection or cell destruction, the DNAs are directly in contact with the cytoplasm, forming a danger signal and inducing innate immune responses (Ablasser and Hur 2020; Motwani et al. 2019). DNAs contained in T-MPs are known to be sensed via the cGAS-STING signaling pathway, which cause the phosphorylation of STAT6 (Ma et al. 2016a; b; Zhang et al. 2015). In this research, we found that knockdown of cGAS, STING, TBK1, or STAT6 by siRNA can consistently abrogate IRF4 upregulation in monocytes which have endocytosed T-MPs, demonstrating that DNAs contained in T-MPs induce IRF4 upregulation by activating the cGAS-STING-STAT6 signaling axis.

A significant discovery in this research is that T-MP-loaded monocytes can treat tumors. We have shown that monocytes could readily take up T-MPs, which lead to presentation of tumor antigens and specific proliferation of T lymphocytes. Thus, we assumed that T-MP-loaded monocytes may achieve therapeutic effects. Our results showed that T-MP-loaded monocytes can treat tumors though stimulating specific proliferation of T lymphocytes, which provide a new approach for oncotherapy.

## Conclusion

This study not only highlights the crucial role of monocytes in tumor prevention via T-MPs, but also reveals the mechanism by which T-MPs induce monocytes to differentiate into moDCs. In addition, our findings show T-MP-loaded monocytes also exhibit therapeutic effects in models of primary tumors and tumor metastasis. These promising results might provide novel vaccination strategy for the development of tumor vaccines and facilitate the application of T-MPs for clinic oncotherapy.

## Materials and methods

### Mice and cell lines

Female 6-week-old WT BALB/c mice, WT C57BL/6 mice and BALB/c-nude mice were purchased from the Experimental Animal Center of Zhengzhou University (Zhengzhou, China). CCR2^−/−^ mice were donated by L. Gao's laboratory (Huazhong University of Science and Technology, Wuhan, China). OT-1 TCR-transgenic mice (C57BL/6-Tg (TcraTcrb) 1100Mjb/J) were donated by B. Huang's laboratory (Huazhong University of Science and Technology, Wuhan, China). All experiments involving mice were approved by the Biological and Medical Ethics Committee of Xinxiang University. Mouse H22 hepatocarcinoma, B16-F10 melanoma, CT26 colon carcinoma and B16-OVA melanoma cell lines were obtained from the China Centre for Type Culture Collection (CCTCC), and cultured in accordance with the guidelines of the manufacturer.

### Collection of T-MPs

Tumor cells were irradiated under UV light for 1.5 h and cultured in a CO_2_ incubator for 24 h. Next, the supernatants were collected for T-MPs isolation. Briefly, the supernatants were centrifuged for 10 min at 1000×*g* to eliminate whole cells and then centrifuged for 2 min at 14,000×*g* to eliminate debris. Finally, the supernatants were centrifuged for 1 h at 14,000×*g* to collect T-MPs. T-MPs were washed 4 times with PBS and then resuspended in suitable volume of PBS. The concentration and particle size of T-MPs were measured by a NTA system (NanoSight NS300, Malvern Panalytical).

### Immunization and tumor challenge

#### Prophylactic experiments

BALB/c mice were intramuscularly inoculated 3 times with 2 × 10^7^ H22-MPs or PBS into the right rear thigh on days -14, -13, and -7. Next, mice were subjected to 3 × 10^5^ H22 hepatocarcinoma cells into the right rear thigh via i.m. injection on day 0. For B16-F10 melanoma models, C57BL/6 mice or CCR2^−/−^ mice were subjected to 5 × 10^5^ B16-F10 melanoma cells via i.m. injection after preventive inoculation with 2 × 10^7^ B16-MPs 3 times following aforementioned method. For immune-deficient models, BALB/c-nude mice were intramuscularly inoculated with 2 × 10^7^ H22-MPs or PBS into the right rear thigh and then intramuscularly injected with 3 × 10^5^ H22 hepatocarcinoma cells.

#### Therapeutic experiments

BALB/c mice were subjected to 3 × 10^5^ H22 hepatocarcinoma cells into the right rear thigh via i.m. injection. H22-MPs were cultured with BALB/c mouse monocytes for 12 h and used as H22-MP-loaded monocytes. 5 days after inoculation of tumor cells, mice with tumor were vaccinated with 1 × 10^6^ H22-MP-loaded monocytes though intravenous injection. Mice in control group received PBS or unpulsed monocytes. Finally, the tumor weight was measured on day 10. C57BL/6 mice with B16-F10 melanomas were treated similarly. In tumor metastasis models, C57BL/6 mice were subjected to 1 × 10^6^ B16-OVA melanoma cells through i.m. injection, and then vaccinated using 1 × 10^6^ OVA-MP-loaded monocytes or PBS through tail vein 3 times, once every 5 days. The black B16-OVA tumor nodules in the lungs were calculated after 21 days.

### Isolation of mouse monocytes from mouse bone marrow

The femurs and tibias of two hind legs of mouse were isolated, and the bone marrow cells were collected. Monocytes were isolated according to the instructions of Monocyte Isolation Kit (Miltenyi Biotec, Cat#: 130-100-629). Briefly, bone marrow cells were mixed with FcR blocking reagent. Then cocktail of biotin-conjugated monoclonal antibodies were added into bone marrow cells, which were incubated for 5 min. Next, cells were washed by labeling buffer. And then Anti-Biotin magnetic beads were mixed thoroughly with cells and incubated for 10 min. At last, the single-cell suspension was passed through a magnetic column, and the cells in the flow-down liquid were collected, which were bone marrow-derived monocytes.

### CCR2^−/−^ bone marrow cells transplantation

Femurs and tibias of WT C57BL/6 mice and CCR2^−/−^ mice were harvested, and bone marrow cells were collected. Recipient C57BL/6 mice were subjected to 9 Gy of irradiation and then transplanted with 5 × 10^6^ bone marrow cells derived from WT C57BL/6 or CCR2^−/−^ mice. After 1 month, these mice were used for subsequent experiments.

### Flow cytometric analysis and cell isolation

For phenotypic analysis of immunocyte in muscle, muscles around the femur were collected, cut into 1–2 mm^2^ pieces, and digested at 37 ℃ for 1 h with hyaluronidase (MACKLIN, Cat#: 37259-53-3), collagenase II (Solarbio, Cat#: C8150) and DNase I (Solarbio, Cat#: D8071). Single-cell suspensions were washed in PBS and incubated with the following surface antibodies: CD45 (Invitrogen, Cat#: 64-0451-82), CD11b (Invitrogen, Cat#: 11-0112-82), Ly6C (Invitrogen, Cat#: 17-5932-82), F4/80 (Invitrogen, Cat#: 17-4801-82), CD11c (Invitrogen, Cat#: 17-0114-82), MHCII (Invitrogen, Cat#: 11-5321-81). To examine the number of moDCs in dLN, mouse inguinal lymph node was isolated, and ground into a single-cell suspension, which was stained with surface antibodies: CD11c (Invitrogen, Cat#: 12-0114-81), MHCII (Invitrogen, Cat#: 11-5321-81) and CD64 (Invitrogen, Cat#: 17-0641-80). In some cases, cells were stained with CD3 (Invitrogen, Cat#: 11-0031-82), CD8 (Invitrogen, Cat#: 17-0081-82) and OVA257-264 (SIINFEKL) peptide bound to H-2Kb (HelixGen, Cat#: HG08T14028) monoclonal antibodies to analyze the specific proliferation of T lymphocytes which can bind to SIINFEKL antigen peptides.

For intracellular cytokine analysis, cells were incubated with PMA and ionomycin (Invitrogen, Cat#: 00-4975-93) for 4 h, and then stained with CD3 (Invitrogen, Cat#: 11-0031-82) and CD8 (Invitrogen, Cat#: 17-0081-82) antibodies. After surface staining, cells were treated with an intracellular staining kit (Invitrogen, Cat#: 88-8824-00) and stained with IFNγ antibody (Invitrogen, Cat#:12-7311-82).

For collection of macrophages from muscles, cell suspensions from muscles were stained with CD11b (Invitrogen, Cat#: 11-0112-82) and F4/80 (Invitrogen, Cat#: 17-4801-82) antibodies. For collection of monocytes from muscles, cell suspensions from muscles were stained with CD11b (Invitrogen, Cat#: 11-0112-82) and Ly6C (Invitrogen, Cat#: 17-5932-82) antibodies. For collection of moDCs from dLN, cell suspensions of dLN were labelled with CD11c (Invitrogen, Cat#: 12-0114-81), MHCII (Invitrogen, Cat#: 11-5321-81) and CD64 (Invitrogen, Cat#: 17-0641-80) flow antibodies. And then cell sorting were carried out by a cell sorter (BD FACSAria III, BD Biosciences).

### Gene silencing assay

siRNAs which target mouse *TLR9* (siRNA#1: GCCUCUCCUUGAUCUCCAA; siRNA#2: CCAUCUGUCUCUGAAGUAU), mouse *IRF4* (siRNA#1: GAAUUGUUUAAAGGCAAGU; siRNA#2: GUCAUUCUUCCAUCCAAGA), mouse *cGAS* (GCAGCUACUAUGAACAUGU), mouse *STING* (GGAGCCGAAGACUGUACAU), mouse *TBK1* (GGAAGUGUCCAAGUAUCAA), mouse *STAT6* (GCUGAUCAUUGGCUUUAUU) and control siRNAs were obtained from RiboBio. Using Lipofectamine RNAiMAX (Invitrogen, Cat#: 13778075), siRNAs were transfected into macrophages or monocytes.

### Real-time PCR

RNAs were extracted from cells with RNA-Quick Purification Kit (YISHAN, Cat#: ES-RN001). RNA to cDNA conversion was carried out using the Fast All-in-One RT Kit (YISHAN, Cat#: ES-RT001). Real-time PCR analyses were carried out by a Super SYBR Green qPCR Master Mix (YISHAN, Cat#: ES-QP002). Housekeeping gene β-actin were used to normalize the mRNA levels. Primer pairs targeting genes are shown as follows:mouse IRF4 forward (5′-GAACGAGGAGAAGAGCGTCTTC-3′);mouse IRF4 reverse (5′-GTAGGAGGATCTGGCTTGTCGA-3′);mouse TLR9 forward (5′-GCTGTCAATGGCTCTCAGTTCC-3′);mouse TLR9 reverse (5′-CCTGCAACTGTGGTAGCTCACT-3′);mouse cGAS forward (5′-ACATGTGAAGATTTCTGCTCCT-3′);mouse cGAS reverse (5′-AGAAATGACTCAGCGGATTTCC-3′);mouse STING forward (5′-ATTGTCTACCAAGAACCCACAG-3′);mouse STING reverse (5′-ATCCATACCACTGATGAGGAG-3′);mouse TBK1 forward (5′-TAGTCTTTCTCAGGGTCTTCAGG-3′);mouse TBK1 reverse (5′-AAGCACATCACTGGTCTCTG-3′);mouse STAT6 forward (5′-GTCACTATAAGCCCGAACAG-3′);mouse STAT6 reverse (5′-GCCATTCCAAGATCATAAGGT-3′);mouse β-actin forward (5′-CATTGCTGACAGGATGCAGAAGG-3′);mouse β-actin reverse (5′-TGCTGGAAGGTGGACAGTGAGG-3′).

### Preparation of mouse BMDMs

Femurs and tibias of mouse were harvested, and bone marrow cells were collected which were cultured with recombinant mouse MCSF (20 ng/ml, PeproTech, Cat#: 315-02-10) in BASIC RPMI 1640 medium (Gibco, Cat#: C22400500BT) containing 10% FBS (Gibco, Cat#: 10100147C) for 5 days. The medium was changed every 48 h, and after 5 days of culture, the macrophages were used for subsequent experiments.

### Migration assays

In vitro cell migration assays were performed using trans-well chambers (Corning, Cat#: 3421). 3 × 10^5^ mouse BMDMs were cultured in a 24-well plate, and 1 × 10^5^ monocytes collected from mouse bone marrow cells were cultured in a trans-well chamber. After 48 h, the trans-well chambers were fixed for 30 min with methanol, then stained for 15 min with Giemsa dye solution. Images of five different 400-fold fields were captured from each trans-well chamber and the number of migratory cells was counted.

### Two-photon confocal microscopy

To detect which type of immune cells endocytosed T-MPs in the muscle, PKH26 (Sigma-Aldrich, Cat#: MINI26) stained T-MPs were injected into the biceps femoris muscle of the right hind leg of mice. After 24 h, the muscles around femur were isolated, embedded in Tissue-Tek OCT, and frozen in dry ice. OCT tissue frozen sections were stained with CD11b (Invitrogen, Cat#: 14-0112-82), Ly6C (Invitrogen, Cat#: MA1-81899), F4/80 (Invitrogen, Cat#: 14-4801-82), CD11c (Invitrogen, Cat#: 14-0114-82) or MHCII (Invitrogen, Cat#: 14-5321-82) antibodies at 4 ℃ overnight. Then the samples were washed 4 times by PBS and labeled with secondary fluorescent antibodies at room temperature for 60 min. Next, the sections were stained in DAPI solution. Finally, the images were visualized by a two-photon confocal microscope. In some cases, H22-MPs were labeled with PKH26 or PKH67 (Sigma-Aldrich, Cat#: MINI67). Mouse monocytes were cultured with PKH26 or PKH67-labeled H22-MPs. After 14 h, the monocytes were analyzed with ER, Golgi, mitochondria, endosome or lysosome trackers by a two-photon confocal microscope.

### Western blot

Monocytes or T-MPs lysate proteins were measured using IRF4 (Cell Signaling Technology, Cat#: 62834), TBK1 (Cell Signaling Technology, Cat#: 38066), STAT6 (Cell Signaling Technology, Cat#: 5397), Phospho-TBK1 (Cell Signaling Technology, Cat#: 5483), Phospho-STAT6 (Cell Signaling Technology, Cat#: 56554), β-actin (Proteintech, Cat#: 81115-1-RR), and then incubated with HRP-conjugated secondary antibodies. Finally, chemiluminescent detection was used to analyze protein expression.

### Statistical analysis

Statistical analysis and graphs were carried out by GraphPad Prism 7 software. Results are represented by mean ± s.e.m. and two-tailed Student's *t* test was used to statistically analyze. Kaplan–Meier survival curves were statistically analyzed by Log-rank test. A *P-*value of less than 0.05 was considered as statistically significant.

## Supplementary Information

**Additional file 1: Fig. S1.** Intramuscular injection of B16-MPs inhibited tumorigenesis of B16-F10 melanoma. **Fig. S2.** Intramuscular inoculation of MPs from CT26 colon carcinoma cells rather than those from H22 hepatocarcinoma cells led to prevention of CT26 tumor growth. **Fig. S3.** T-MPs as a tumor vaccine presented a good safety. **Fig. S4.** B lymphocytes and T lymphocytes in BALB/c mice don't endocytose T-MPs. **Fig. S5.** T-MPs are mainly endocytosed by monocytes and macrophages in C57BL/6 mice. **Fig. S6.** T-MPs treatment doesn't cause the proliferation of moDCs in the dLN. **Fig. S7.** DNAs and RNAs were isolated from T-MPs.

## Data Availability

The data and materials supporting this study are available from the corresponding author under reasonable request.

## Abbreviations

MPs: Microparticles
EVs: Extracellular vesicles
APCs: Antigen-presenting cells
T-MPs: Tumor microparticles
DCs: Dendritic cells
cDCs: Classical DCs
moDCs: Monocyte-derived DCs
dLN: Draining lymph node
s.c.: Subcutaneous
i.m.: Intramuscular
H22-MPs: H22 hepatocarcinoma cell-derived MPs
B16-MPs: B16-F10 melanoma cell-derived MPs
CT26-MPs: CT26 colon carcinoma cell-derived MPs
OVA-MPs: B16-OVA melanoma cell-derived MPs
NTA: Nanoparticle tracking analysis
BMDMs: Bone marrow-derived macrophages
WT: Wild-type
2-DG: 2-Deoxy-d-glucose
TLR9: Toll-like receptor 9
