# Supplementary material for: Monocytes reprogrammed by tumor microparticle vaccine inhibit tumorigenesis and tumor development
Source: Cancer Nanotechnol. 2023 Apr 17;14(1):34. doi: 10.1186/s12645-023-00190-x (PMC10106871; doi:10.1186/s12645-023-00190-x)
Supplement: Supplementary file 1 — Additional file 1: Fig. S1. Intramuscular injection of B16-MPs inhibited tumorigenesis of B16-F10 melanoma. Fig. S2. Intramuscular inoculation of MPs from CT26 colon carcinoma cells rather than those from H22 hepatocarcinoma cells led to prevention of CT26 tumor growth. Fig. S3. T-MPs as a tumor vaccine presented a good safety. Fig. S4. B lymphocytes and T lymphocytes in BALB/c mice don't endocytose T-MPs. Fig. S5. T-MPs are mainly endocytosed by monocytes and macrophages in C57BL/6 mice. Fig. S6. T-MPs treatment doesn't cause the proliferation of moDCs in the dLN. Fig. S7. DNAs and RNAs were isolated from T-MPs. [file 12645_2023_190_MOESM1_ESM.docx]

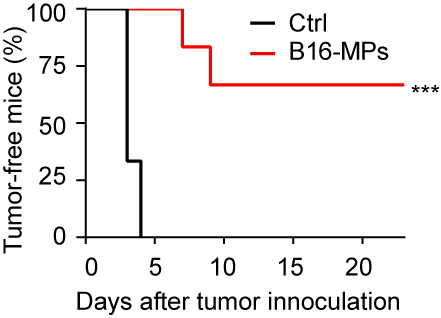


# Supplementary Fig. S1. Intramuscular injection of B16-MPs inhibited tumorigenesis of B16-F10 melanoma. C57BL/6 mice (*n* = 6), intramuscularly inoculated with PBS or B16-MPs on days -14, -13, and -7, were given i.m. injection of 5×10^5^ B16-F10 melanoma cells into the right rear thigh muscle on day 0. Kaplan-Meier analysis was used to assess the percentage of mice which were tumor-free. *P* value was calculated using Log-rank test. ****P* < 0.001.


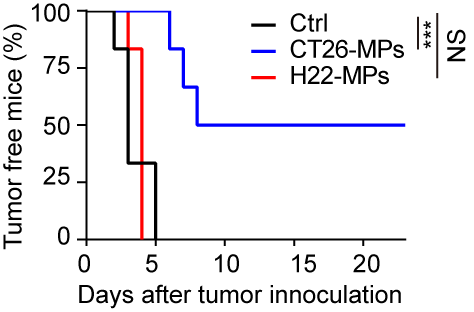


# Supplementary Fig. S2. Intramuscular inoculation of MPs from CT26 colon carcinoma cells rather than those from H22 hepatocarcinoma cells led to prevention of CT26 tumor growth. BALB/c mice (*n* = 6) were intramuscularly inoculated 3 times with PBS, CT26-MPs or H22-MPs into the right rear thigh on days -14, -13, and -7. Next, mice were subjected to 3×10^5^ CT26 colon carcinoma cells into the right rear thigh via i.m. injection on day 0. Kaplan-Meier analysis was used to assess the percentage of mice which were tumor-free. *P* values were calculated using Log-rank test. ****P* < 0.001; NS, not significant.


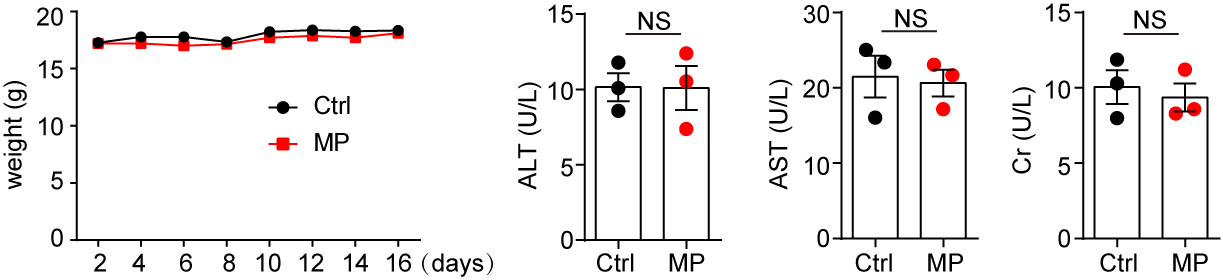


**Supplementary Fig. S3.** T-MPs as a tumor vaccine presented a good safety. BALB/c mice (*n* = 3) were intramuscularly immunized with H22-MPs or PBS on days -7, -6, and 0. Weight of mice was measured over the ensuing day 16. On day 17, the mice were sacrificed, and the serum levels of alanine aminotransferase (ALT), aspartate aminotransferase (AST) and creatinine (Cr) were detected. Mean±s.e.m. is represented in the data and two-tailed unpaired Student's *t* test was used to statistically analyze the *P* values. NS, not significant.


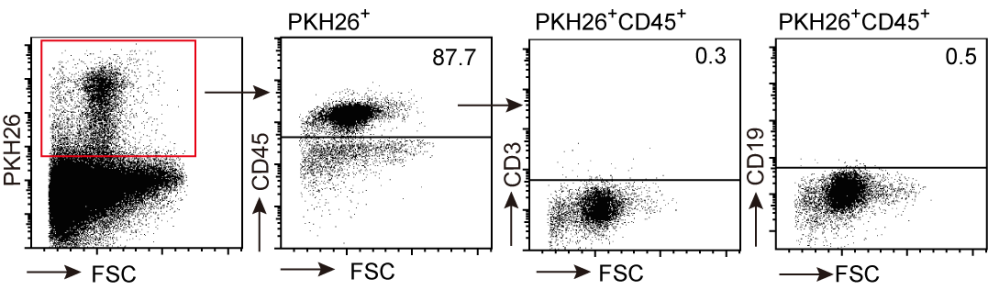


# Supplementary Fig. S4. B lymphocytes and T lymphocytes in BALB/c mice don't endocytose T-MPs. BALB/c mice were treated with PKH26-labeled H22-MPs via i.m. injection. After 24 hours, the expression of CD45 in PKH26 positive cells from thigh muscle was analyzed via flow cytometry. CD3+ T lymphocytes and CD19+ B lymphocytes in PKH26+CD45+ cells were also analyzed by flow cytometry.


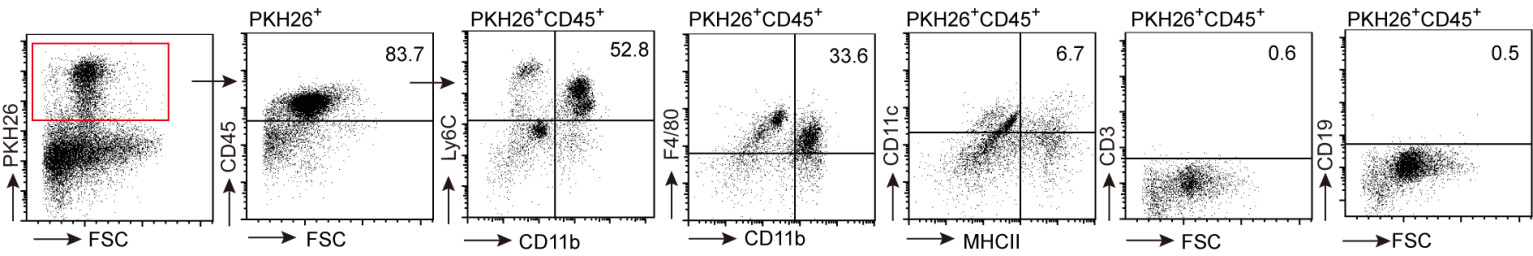


# Supplementary Fig. S5. T-MPs are mainly endocytosed by monocytes and macrophages in C57BL/6 mice. C57BL/6 mice were intramuscularly immunized with PKH26-labeled B16-MPs. After 24 hours, the expression of CD45 in PKH26 positive cells from thigh muscle was analyzed by flow cytometry. CD11b+Ly6C+ monocytes, CD11b+F4/80+ macrophages, CD11c+MHCⅡ+ DCs, CD3+ T lymphocytes and CD19+ B lymphocytes in PKH26+CD45+ cells were also analyzed via flow cytometry.


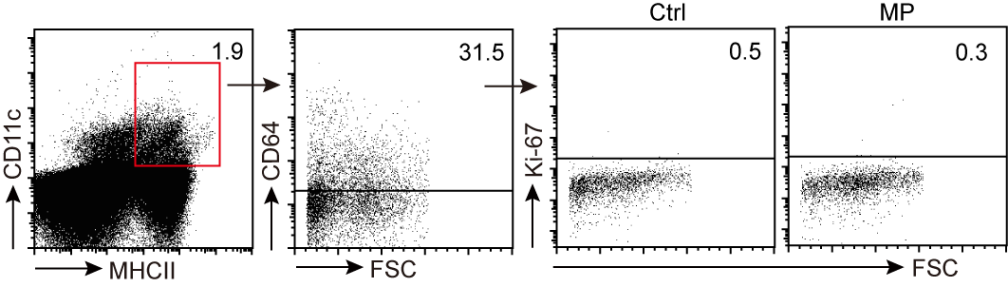


# Supplementary Fig. S6. T-MPs treatment doesn't cause the proliferation of moDCs in the dLN. BALB/c mice were treated with H22-MPs or PBS via i.m. injection. 48 hours later, Ki-67 expression in CD11c^+^MHCⅡ^+^CD64^+^ moDCs was analyzed by flow cytometry.


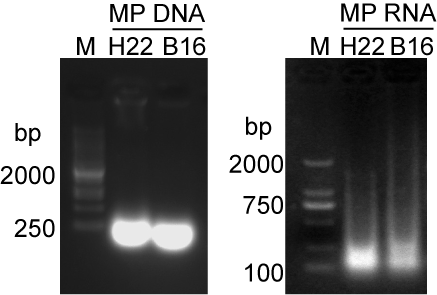


# Supplementary Fig. S7. DNAs and RNAs were isolated from T-MPs. DNAs in H22-MPs or B16-MPs were extracted by DNA extraction kit, and RNAs were extracted with TRIzol reagent.
